# Spermatic Hydrocele

**Published:** 1880-03

**Authors:** 


TRANSLATIONS.

[From Virchow's Handbook of Special Pathology and Therapy, Band VI., Part II.—"Diseases of the Male Generative Organs," by Dr. F. R. Von Pitha, Professor of Surgery in Vienna.]

### SPERMATIC HYDROCELE.

---

TRANSLATED FROM THE GERMAN BY JAMES AUMANN, M. D., TAZEWELL COUNTY, VIRGINIA.

---

The occurrence of spermatozoa in a hydrocele (*H. Spermatica*), though rare, is nevertheless a very interesting fact, about which there can no longer be any doubt, after the observations of Curling, Sedillot, Gosselin, Uhde, and others, which I have already recorded in a previous chapter. I have myself only recently met with two remarkable cases of it.

The first case was that of a strong and perfectly healthy man, 40 years of age, and a cooper by occupation, who consulted me in July, 1854, about a hydrocele, which developed spontaneously, and had already existed for four years. The scrotal tumor was as large as a man's fist, oval in shape, very tense, and distinctly fluctuating; the testicle could be felt on the posterior wall of the tumor: in short, it presented all the characteristics of a common hydrocele of the *tunica vaginalis propria*. On puncturing the tumor, I was surprised at the milk-like, perfectly homogeneous appearance of the contained liquid, which I at once subjected to a careful examination. The microscope revealed the presence of a vast number of spermatozoa, which were in active motion, and in addition spermatic cells, and a few epithelial cells. The fluid had a slightly alkaline reaction, and a specific gravity of 1.0022. On standing for some time a very light, loose and flaky sediment formed, in consequence of which the fluid lost its milky appearance, and resembled moderately clear whey. Chemical analysis showed an abundance of albumen, albuminate of sodium and free sodium, chloride of sodium and saponified fat, with traces of phosphate of calcium. The abundant white sediment consisted solely of the above-named spermatic elements.

The second case was observed shortly after the first. A robust hack-driver, aged 45 years, who had always been healthy, with the exception of a large scrotal tumor, which had existed for thirty years, had in August, 1854, a sudden attack of ischuria, which caused him to come to our clinic. On introducing a catheter it was apparent that the retention of urine was solely due to the large scrotal tumor, which, according to the statements of the patient, had rapidly increased in size during the last week. The tumor named pressed the urethra up against the symphysis in such a manner that the passage of urine became impossible, and even the catheter would enter only with difficulty, and when the precaution was observed to avoid the tumor by a circular sweep. The tumor was as large as a child's head, was heart-shaped, with the flat base directed toward the septum, very tense, distinctly fluctuating, and completely opaque; above, at the external abdominal ring, it was distinctly separated from the free inguinal hernia which extended from that point upward; below, at its rounded apex, the testicle—very distinctly isolated—was situated, which was otherwise similar in every respect to the one on the healthy side. Thus, we had to deal with a *hydrocèla encysté* of unusual size and shape, and, judging from these conditions and the absence of transparency, we at once expressed the probability of its being a *hydrocele cystica spermatica.* But the trocar let out a dark, olive-green, turbid fluid (amounting to about two pounds), which seemed to disprove our surmise. On microscopical examination, however, numerous spermatozoa, with active motion, were found in the fluid. The patient stated that the scrotal tumor, which had previously been small, had suddenly increased in size in consequence of a blow received ten years ago, and had been constantly enlarging since that time. The ischuria disappeared entirely after the cyst was opened. In this case, the unusual shape and size of the cyst; the position of the testicle, below and to one side; the opacity (in connection with very distinct fluctuation), and the sudden enlargement of the tumor after a blow, led from the first to the probable diagnosis of a *hydrocele spermatica*, our attention having been directed to it by the previous case. Without this coincidence, we would, in all likelihood, have failed to discover this remarkable condition. In the first case, on the other hand, it was simply the unexpected, milky contents of the otherwise common hydrocele that forced the diagnosis upon us. All the cases which we have met with since (*circa* 15) were recognized by the shape of the tumors alone.

The question as to how the spermatozoa get into the hydrocele, was, until quite recently, difficult to answer, owing to the absence of direct anatomical researches. At present we are indebted to Luschka's excellent work on "Appendicula Growths of the Testicle" (Virchow's Archiv., Vol. VI., p. 310) for a full explanation of this previously obscure subject. For the formation of the hydrocele in question it is, of course, absolutely necessary that a communication between the respective cavity containing fluid and the seminal tubes should exist at some point. As such a communication could not be observed in the *tunica vaginalis propria*, common hydrocele was very naturally excluded. For this reason, Gosselin will only recognize a *hydrocele spermatica cystica*, and called the case, which came under his observation, a large cyst on the testicle (*Untersuchungen über die pseudomembranäse Vrdkg. der tunic. vagin. bei Hydrocele und Vlœmatok., Arch. gen. Sept., Novbr., Decbr.*, 1851).

In the case of *hydrocele cystica*, the required communication is, in fact, much easier to understand: with the *vas deferens*, the epididymis, the *vasis efferentibus testis*, etc., either by assuming a congenital abnormality (*vas aberrans Halleri—Uhde*), or an acquired perforation, rupture, injury (puncture) of the seminal tubes, as, for example, might have been the case in our second patient. But the first case can by no means be explained on this supposition. Here the tumor presented itself, as regards volume, shape and relation to the testicle—having been examined both before and after puncturing—as a common *hydrocele tunica vaginalis testis*, which had, moreover, formed spontaneously in a perfectly healthy man, who had never suffered from any specific or traumatic affection of his genitals, and which had never been operated for. All investigations in regard to any special modifications in the mode of origin and development of hydroceles have been of no avail.

This case leads, then, to the assumption of the existence of a communication between the *tunica vaginalis* and the seminal tubules of the testicle corresponding to the normal relation of the latter organ to the *tunica vaginalis.* Now, Luschka's excellent researches have not only shown us the possibility of such a communication, but also the mediating organ, in the non-pedunculated hydatid of Morgagni. Beneath the head of the epididymis, corresponding to the anterior end of its inferior border, there is almost invariably found a somewhat rounded vesicle, varying in size from that of a lentil seed to that of a pea, which represents the remains of one of the uppermost blind tubules of the Wollfian body (Kobelt), and is completely surrounded by the visceral layer of the *tunica vaginalis testis*, and the cavity of which almost always communicates freely with the seminal tubules of the epididymis, and, therefore, usually contains spermatic elements.

Now, this non-pedunculated hydatid of Morgagni is, doubtless, the real seat of development of the *hydrocele spermatica*, whether it gradually expands to a considerable size (*hydrocele cystica spermatica*), or bursts in consequence of a rapid accumulation of semen, and discharges its contents into the cavity of the *tunica vaginalis testis*, when the latter is already the seat of an accumulation of fluid (*hydrocele spermatica tunicæ vaginalis propriæ.*) We have just given an example of each of these varieties.

